# COVID-19-related mortality and hospital admissions in the VIVALDI study cohort: October 2020 to March 2023

**DOI:** 10.1016/j.jhin.2023.10.021

**Published:** 2024-01

**Authors:** O. Stirrup, M. Krutikov, B. Azmi, I. Monakhov, A. Hayward, A. Copas, L. Shallcross

**Affiliations:** a Institute for Global Health, University College London, London, UK; b UCL Institute of Health Informatics, London, UK; c UK Health Security Agency, London, UK; d UCL Institute of Epidemiology & Healthcare, London, UK; e Health Data Research UK, London, UK

**Keywords:** Care homes, COVID-19, Infection fatality ratio, Infection hospitalization ratio, SARS-CoV-2

## Abstract

**Background:**

Long-term-care facilities (LTCFs) were heavily affected by COVID-19 early in the pandemic, but the impact of the virus has reduced over time with vaccination campaigns and build-up of immunity from prior infection.

**Objectives:**

To evaluate the mortality and hospital admissions associated with SARS-CoV-2 in LTCFs in England over the course of the VIVALDI study, from October 2020 to March 2023.

**Methods:**

We included residents aged ≥65 years from participating LTCFs who had available follow-up time within the analysis period. We calculated incidence rates (IRs) of COVID-19-linked mortality and hospital admissions per calendar quarter, along with infection fatality ratios (IFRs, within 28 days) and infection hospitalization ratios (IHRs, within 14 days) following positive SARS-CoV-2 test.

**Results:**

A total of 26,286 residents were included, with at least one positive test for SARS-CoV-2 in 8513 (32.4%). The IR of COVID-19-related mortality peaked in the first quarter (Q1) of 2021 at 0.47 per 1000 person-days (1 kpd) (around a third of all deaths), in comparison with 0.10 per 1 kpd for Q1 2023 which had a similar IR of SARS-CoV-2 infections. There was a fall in observed IFR for SARS-CoV-2 infections from 24.9% to 6.7% between these periods, with a fall in IHR from 12.1% to 8.8%. The population had high overall IRs for mortality for each quarter evaluated, corresponding to annual mortality probability of 28.8–41.3%.

**Conclusions:**

Standardized real-time monitoring of hospitalization and mortality following infection in LTCFs could inform policy on the need for non-pharmaceutical interventions to prevent transmission.

## Introduction

Long-term-care facilities (LTCFs) provide nursing and residential care to 410,000 older adults in England, approximately 0.7% of the population [1]. Care home residents are frailer [2] than their community-dwelling peers, the majority are older than 80 years, and average life expectancy from first entry is 1–2 years [3,4]. In 2019, all-cause mortality was estimated to be 10-fold higher in care home residents compared with community-dwelling adults aged >65 years [5]. The pandemic led to a global surge in deaths in residents, who accounted for more than 40% of all COVID-19-related deaths in 22 countries [6].

Most countries responded to the evolving crisis in care homes by introducing non-pharmaceutical interventions (NPIs) to reduce SARS-CoV-2 transmission, such as controlling people's movements and contacts, using personal protective equipment (PPE), and regular testing for SARS-CoV-2 [7]. In England, vaccination against SARS-CoV-2 began in December 2020 [8], and many residents developed hybrid immunity having been infected with SARS-CoV-2 at some point during the pandemic. Vaccination coincided with major declines in SARS-CoV-2-related deaths and hospital admissions in residents [9]. However, routine use of NPIs continued into the third year of the pandemic (2022) [10], largely driven by concerns about high community incidence of SARS-CoV-2 and the threat posed by new SARS-CoV-2 variants. Whether continued use of NPIs was justified is difficult to ascertain, given the lack of evidence on the effectiveness and cost-effectiveness of NPIs in this setting, and the undeniable negative consequences of measures such as visitor restrictions and social isolation on residents' physical and mental health and well-being [11]. From the second half of 2021, antivirals and neutralizing monoclonal antibodies targeting SARS-Cov-2 were approved for use. These are deployed to individuals from high-risk groups to reduce the risk of severe outcomes from infection, but being a care home resident does not currently make someone eligible for these therapies in the UK.

We hypothesized that detailed analysis of mortality incidence rates (IRs) and infection fatality ratios (IFRs), along with hospital admission rates and infection hospitalization ratios (IHRs), could provide insights into how the impact of infection in residents changed over successive waves of the pandemic in England.

The aims of this study were to evaluate the overall impact of COVID-19 on mortality rates of older care home residents in the VIVALDI study cohort, and to investigate how the IFR for SARS-CoV-2 infections has changed over time in this population.

## Methods

The analysis period was defined as 1^st^ October 2020 to 31^st^ March 2023, corresponding to the period in which a national programme of widespread testing had become implemented in LTCFs in England, up until 12 months after cessation of regular asymptomatic testing in residents on 31^st^ March 2022. During the period of regular testing, residents were tested monthly with more frequent testing in outbreaks or if symptoms occurred. Linkage of lateral flow device (LFD)/polymerase chain reaction (PCR) tests to LTCFs enabled identification of residents of participating facilities. Residents aged ≥65 years were eligible for inclusion if they had at least one PCR or LFD test result within the analysis period, or within 90 days prior to the start of this period, linked to an LTCF participating in the VIVALDI study [12,13]. Residents were excluded from the analysis if they were missing any dates of vaccinations received, or if they died on the date of first recorded SARS-CoV-2 test within the VIVALDI study.

We retrieved all available PCR and LFD results from the national testing programme through the COVID-19 Datastore [12]. Test results and vaccination, mortality and hospitalization data from national records were linked to study participants using pseudo-identifiers based on National Health Service (NHS) numbers. The legal basis to access data is provided by Health Research Authority Confidentiality Advisory Group approval (21/CAG/0156). Ethical approval was obtained from South Central-Hampshire B Research Ethics Committee (20/SC/0238).

Residents were considered to be under follow-up from the latest of: first recorded PCR or LFD test within a participating care home or 1^st^ October 2020. Follow-up ended at the earliest of: 90 days after last test within a participating care home if prior to 1^st^ January 2022, 31^st^ March 2023 if last test recorded on or after 1^st^ January 2022, or date of death. Residents with any test recorded in 2022 were therefore considered to be under follow-up until the end of March 2023 unless they died before this, as most individuals remain within LTCFs once admitted; this was decided because of the cessation of regular asymptomatic testing at the end of March 2022.

COVID-19-related mortality was defined as any death within 28 days of a positive test for SARS-CoV-2 or with confirmed COVID-19 recorded as a primary or any secondary cause of death on the death certificate. We estimated the IR of total mortality, COVID-19-related mortality and non-COVID-19 mortality per calendar quarter. These IRs were used to calculate the implied annualized mortality rates to provide an estimate of mortality risk for an individual followed up for one year. We also calculated the implied probability of non-COVID-19 mortality within a 28-day period, for comparison with mortality in the 28 days following a detected SARS-CoV-2 infection. COVID-19-linked hospitalization was defined as any hospital admission with positive SARS-CoV-2 test in the preceding 14 days or one day after, or with COVID-19 recorded as the primary or any secondary ICD10 code for the admission. IRs per quarter and annualized cumulative incidence were estimated for any COVID-19-linked hospitalization and for those with COVID-19 as the primary admission code.

A positive test was considered to define a new SARS-CoV-2 infection if it was more than 30 days after any previous positive tests. This cut-off was chosen to allow relatively rapid reinfection with the emergence of new variants such as Omicron in December 2021 [14]. We estimated the overall combined IR of new and repeat SARS-CoV-2 infections per calendar quarter, and also plotted the daily IR of new and repeat infections over time. We calculated the crude IFR for all mortality within 28 days of a positive test for SARS-CoV-2 infections detected within each calendar quarter, and the crude IHR for all hospital admissions with 14 days of a positive test. Calculations of IFR and IHR included both first recorded and repeat SARS-CoV-2 infections for each resident. Additional deaths and hospitalization data were used from April 2023 to enable complete 28-day and 14-day follow-up for infections detected in March 2023.

Separate Cox regression models were used to investigate risk factors for mortality within 28 days and hospitalization within 14 days of a positive SARS-CoV-2 test. These models included adjustment for age (linear term), sex, calendar quarter at time of infection, previously detected infection (based on combined PCR and LFD results, hospital admission records and anti-nucleocapsid antibody results where available) and vaccination status defined by the following groups: no vaccination recorded, single vaccine dose received, two to 12 weeks since second dose, 12–24 weeks since second dose, 24+ weeks since second dose, two to 12 weeks since most recent booster vaccination, 12–24 weeks since most recent booster and 24+ weeks since most recent booster. A random effect frailty term was included to allow correlation of outcomes within each LTCF. An analysis model was also fitted including an additional separate variable recording whether each person had received a bivalent SARS-CoV-2 vaccine prior to the positive test. Analyses were conducted using Stata v18.0.

## Results

A total of 26,286 residents from 327 LTCFs were included in the analysis (Supplementary Figure S1), of whom there was at least one positive test result for SARS-CoV-2 in 8513 (32.4%). The median age of residents was 86.1 years (interquartile range 79.6–91.3) and 17217 (65.5%) were female. The number of residents considered to be under follow-up varied over time within the analysis period from around 10,000 to 11,500 (Supplementary Figure S2), influenced by multiple factors including: testing policy for SARS-CoV-2 (as test records were required to identify residents within participating homes), resident mortality and the admission of new residents to participating homes.

**Figure 1:**
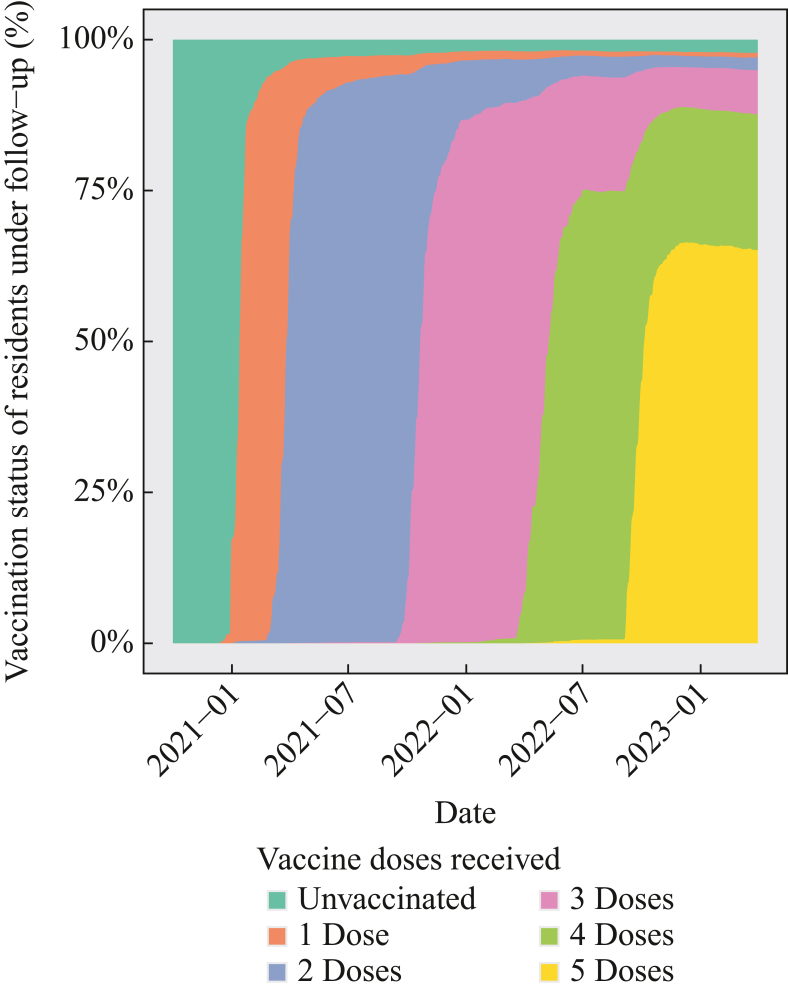
Plot of vaccination status of residents of long-term-care facilities within the VIVALDI study cohort included in the analysis. The percentage of residents with each number of vaccine doses is given among those in follow-up for the analysis at any given point in time.

There was high uptake among residents of primary vaccination against SARS-CoV-2 starting in December 2020 and booster vaccination from September 2021 (Figure 1). There was widespread rollout of fourth- and fifth-dose vaccination from March 2022 and September 2022, respectively, with uptake over 60% for each of these.

**Figure 2:**
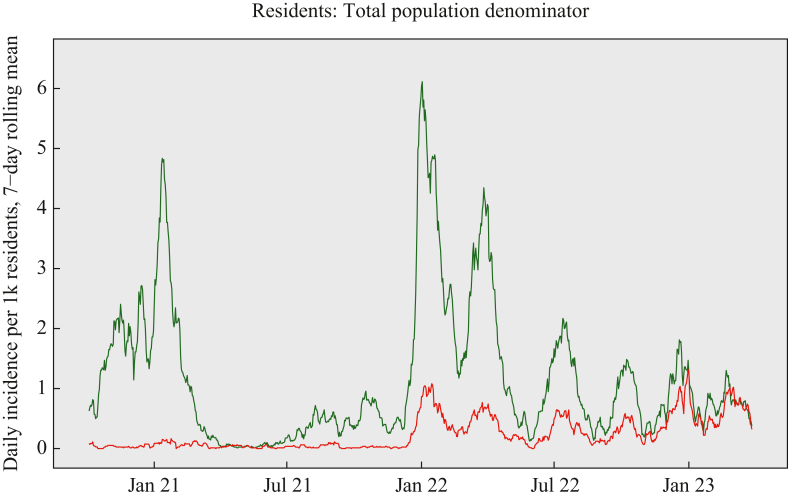
Rolling seven-day average incidence rate of new SARS-CoV-2 infections (green) and repeat SARS-CoV-2 infections (red) among residents of long-term-care facilities in the VIVALDI study. Incidence rates are calculated according to the total population under follow-up.

Over the analysis period considered, there was a first peak of SARS-CoV-2 infections in January 2021 corresponding to spread of the Alpha variant in the UK (Figure 2). The incidence then dropped to very low levels in Spring 2021 following implementation of national lockdown policies. Incidence showed a modest increase in Autumn 2021 before increasing sharply in January 2022 following spread of the Omicron variant, when the rate of re-infections first rose. IRs of primary infections and re-infections then followed a series of peaks and troughs throughout the remainder of 2022 through to March 2023, mirroring national trends and reflecting the lack of national social distancing or lockdown policies in this period.

The incidence of COVID-19-related mortality showed peaks in the first quarter (Q1) of 2021 and Q1 of 2022 (Supplementary Figure S3). Averaged over calendar quarter, the incidence of SARS-CoV-2 was substantially higher in Q1 2022 (2.90 per 1000 person-days (1 kpd)) than in Q1 2021 (1.05 per 1 kpd) (Table I). However, the IR of COVID-19-related mortality peaked in Q1 2021 at 0.47 per 1 kpd (around a third of all deaths), in comparison with 0.22 per 1 kpd for Q1 2022. The relatively low IR of COVID-19-related mortality in the latter period is explained by a fall in the observed IFR for SARS-CoV-2 infections from 24.9% to 6.9%. The population had high overall IRs for mortality for each quarter evaluated, corresponding to estimates of annual mortality probability of 28.8–41.3% for residents in the cohort. Estimates of the background rate of non-COVID-19-related mortality over a 28-day period were in the range 2.3–3.3%, indicating that this should also be considered when evaluating the IFR for SARS-CoV-2 infections in this population.

**Table I:** Summary of non-COVID- and COVID-related mortality among residents of long-term-care facilities in the VIVALDI study

Quarter	Total mortality		Non-COVID mortality			COVID-linked mortality		SARS-CoV-2 incidence^a^	
	IR (per 1 kpd) (95% CI)	Annualized mortality (%)	IR (per 1 kpd) (95% CI)	Annualized mortality (%)	28-Day mortality (%)	IR (per 1 kpd) (95% CI)	Annualized mortality (%)	IR (per 1 kpd) (95% CI)	28- Day IFR (%, *n/N*)
Q4 2020	1.07 (1.01–1.14)	32.5	0.81 (0.76–0.87)	24.6	2.3	0.26 (0.23–0.29)	7.9	0.97 (0.91–1.03)	21.2 (213/1004)
Q1 2021	1.46 (1.38–1.53)	41.3	0.99 (0.93–1.06)	28.1	2.7	0.47 (0.43–0.51)	13.2	1.05 (0.99–1.12)	24.9 (277/1113)
Q2 2021	0.93 (0.87–0.99)	28.8	0.91 (0.86–0.98)	28.3	2.5	0.02 (0.01–0.03)	0.5	0.04 (0.03–0.05)	20.5 (8/39)
Q3 2021	1.13 (1.06–1.19)	33.7	1.09 (1.03–1.16)	32.7	3.0	0.03 (0.02–0.05)	1.0	0.25 (0.22–0.29)	8.7 (25/289)
Q4 2021	1.27 (1.21–1.34)	37.2	1.21 (1.14–1.28)	35.3	3.3	0.07 (0.05–0.08)	1.9	0.74 (0.69–0.79)	8.2 (64/776)
Q1 2022	1.30 (1.23–1.38)	37.9	1.09 (1.02–1.15)	31.6	3.0	0.22 (0.19–0.25)	6.3	2.90 (2.79–3.01)	6.9 (185/2692)
Q2 2022	1.13 (1.07–1.20)	33.9	1.04 (0.97–1.10)	31.0	2.9	0.10 (0.08–0.12)	2.9	0.80 (0.75–0.86)	7.7 (62/807)
Q3 2022	1.10 (1.03–1.16)	33.0	1.01 (0.95–1.08)	30.5	2.8	0.08 (0.06–0.10)	2.4	0.74 (0.69–0.80)	8.1 (62/769)
Q4 2022	1.31 (1.24–1.38)	38.0	1.22 (1.15–1.29)	35.3	3.3	0.09 (0.08–0.11)	2.7	0.97 (0.91–1.04)	6.5 (67/1027)
Q1 2023	1.23 (1.16–1.30)	36.2	1.13 (1.07–1.20)	33.3	3.1	0.10 (0.08–0.12)	2.8	1.02 (0.96–1.09)	6.7 (69/1033)

Incidence rates (IRs) are calculated according to the total population under follow-up at any given point in time, and the implied annualized mortality rates are calculated to provide an estimate of mortality risk for an individual followed up for one year. The IR for detected SARS-CoV-2 infections (new and repeat) is also reported, along with the crude 28-day infection fatality ratio (IFR). CI, confidence interval. 1 kpd, 1000 person-days.

a Infections detected at an individual's first recorded polymerase chain reaction (PCR) or lateral flow device (LFD) test contribute to estimation of the IFR but not SARS-CoV-2 IR (*n*=711), both include new and repeat infections.

The IR for any COVID-19-related hospital admissions also showed peaks in Q1 2021 (0.29 per 1 kpd) and Q1 2022 (0.32 per 1 kpd), but did not show as substantial a drop as mortality in the remainder of 2022 (Table II). When only considering admissions with COVID-19 as the primary ICD-10 code, there was a lower peak in Q1 2022 (0.08 per 1 kpd) in comparison with Q1 2021 (0.14 per 1 kpd).

**Table II:** Summary of any COVID-linked hospital admission and primary COVID hospital admission among residents of long-term-care facilities in the VIVALDI study

Quarter	Any COVID-linked hospital admission		Hospital admission with COVID as primary cause		IHR (%, *n/N*)
	IR (per 1 kpd) (95% CI)	Annual cumulative incidence (%)	IR (per 1 kpd) (95% CI)	Annual cumulative incidence (%)	
Q4 2020	0.27 (0.24–0.31)	9.5	0.13 (0.11–0.16)	4.8	13.9 (140/1004)
Q1 2021	0.29 (0.26–0.32)	10.0	0.14 (0.12–0.17)	5.0	12.1 (135/1113)
Q2 2021	0.02 (0.01–0.03)	0.8	0.01 (0.00–0.01)	0.3	17.9 (7/39)
Q3 2021	0.06 (0.05–0.08)	2.2	0.02 (0.02–0.04)	0.9	14.9 (43/289)
Q4 2021	0.14 (0.12–0.16)	4.9	0.05 (0.04–0.07)	1.9	10.3 (80/776)
Q1 2022	0.32 (0.29–0.36)	11.1	0.08 (0.07–0.10)	3.0	6.8 (184/2692)
Q2 2022	0.19 (0.16–0.22)	6.6	0.03 (0.02–0.05)	1.2	11.8 (95/807)
Q3 2022	0.19 (0.16–0.22)	6.7	0.04 (0.03–0.06)	1.4	12.5 (96/769)
Q4 2022	0.15 (0.12–0.17)	5.2	0.05 (0.04–0.07)	1.9	7.8 (80/1027)
Q1 2023	0.19 (0.16–0.22)	6.6	0.06 (0.05–0.08)	2.3	8.8 (91/1033)

Incidence rates (IRs) are calculated according to the total population under follow-up at any given point in time, and the implied annual cumulative incidence rates are calculated to provide an estimate of the risk for an individual followed up for one year. The 14-day infection hospitalization ratio (IHR) is also presented, based on recorded positive SARS-CoV-2 tests. CI, confidence interval; 1 kpd, 1000 person-days.

Mortality within 28 days of detected SARS-CoV-2 infection was strongly predicted by age (HR 1.06, 95% confidence interval 1.05–1.07, per year) and male sex (1.60, 1.40–1.82) (Table III). Primary two-dose vaccination showed a protective effect against mortality, that waned from two to 12 weeks (0.27, 0.10–0.73) to 12–24 weeks (0.49, 0.26–0.91) and 24+ weeks (0.62, 0.41–0.94) after the second dose. Booster vaccination restored the protective effect, with a lesser degree of waning from two to 12 weeks (0.37, 0.25–0.54) to 12–24 weeks (0.38, 0.26–0.54) and 24+ weeks (0.43, 0.28–0.65) after booster dose. Known previous SARS-CoV-2 infection was associated with a lower risk of mortality (0.69, 0.56–0.84). There were differences in IFR between calendar periods, conditional on the other variables included in the model. The IFR was highest in the first half of 2021, before dropping to a consistent lower level. This could be explained by a combination of multiple factors including dominant SARS-CoV-2 variant, unrecorded prior infections in our population, changes to medical treatment and management and variations in healthcare capacity. A second model was fitted that included a separate variable recording whether each person had received a bivalent SARS-CoV-2 vaccine prior to positive test, this indicated inconclusive evidence of a further protective effect against mortality (0.73, 0.50–1.06). A similar pattern of associations was found in the analysis of hospital admission in the 14 days following a positive test (Supplementary Table S1), although age was not predictive of admission and the protective effect of prior infection was weaker.

**Table III:** Results of Cox regression analyses of mortality in the 28 days following a positive polymerase chain reaction (PCR) or lateral flow device (LFD) test for SARS-CoV-2

	Model 1, HR (95% CI)	Model 2, HR (95% CI)
Age (years)	1.06 (1.05–1.07)	1.06 (1.05–1.07)
Female	Ref	Ref
Male	1.60 (1.40–1.82)	1.59 (1.40–1.82)
Calendar period		
Q4 2020	Ref	Ref
Q1 2021	1.41 (1.16–1.72)	1.41 (1.16–1.72)
Q2 2021	3.37 (1.52–7.46)	3.33 (1.50–7.40)
Q3 2021	0.78 (0.41–1.47)	0.76 (0.40–1.44)
Q4 2021	0.80 (0.52–1.24)	0.77 (0.50–1.20)
Q1 2022	0.80 (0.54–1.17)	0.76 (0.51–1.12)
Q2 2022	0.93 (0.59–1.45)	0.90 (0.57–1.40)
Q3 2022	1.02 (0.65–1.59)	0.98 (0.63–1.54)
Q4 2022	0.88 (0.56–1.37)	1.03 (0.64–1.65)
Q1 2023	0.91 (0.58–1.42)	1.09 (0.67–1.79)
Vaccination status		
None	Ref	Ref
Single vaccine dose	0.53 (0.40–0.69)	0.53 (0.40–0.69)
2–12 Weeks since D2	0.27 (0.10–0.73)	0.28 (0.10–0.74)
12–24 Weeks since D2	0.49 (0.26–0.91)	0.50 (0.26–0.94)
24+ Weeks since D2	0.62 (0.41–0.94)	0.64 (0.42–0.97)
2–12 Weeks since booster	0.37 (0.25–0.54)	0.39 (0.26–0.59)
12–24 Weeks since booster	0.38 (0.26–0.54)	0.40 (0.28–0.58)
24+ Weeks since boost	0.43 (0.28–0.65)	0.43 (0.28–0.65)
Prior infection	0.69 (0.56–0.84)	0.69 (0.56–0.85)
Bivalent vaccine received		0.73 (0.50–1.06)

Both models include a variable describing vaccination status, broken down by time since second dose or most recent booster dose received at point of positive test. Model 2 also includes a separate variable recording whether each person had received a bivalent SARS-CoV-2 vaccine prior to positive test. HR, hazard ratio.

## Discussion

Over the two and a half years of the pandemic assessed in this study, the annual risk of death in care home residents from any cause ranged between 28.8% and 41.3%. The IR of COVID-19-related deaths and hospitalizations peaked in the first quarters of 2021 and 2022, coinciding, respectively, with the emergence of the Alpha and Omicron variants. The proportion of all deaths that were associated with SARS-CoV-2 infection declined substantially over this period, primarily due to high uptake of primary and booster vaccinations [9,15], but the IFR stabilized at a non-negligible level of around 7% in 2022. Our findings highlight the challenges associated with monitoring the impact of infection-linked mortality in care home residents, in view of the high baseline mortality, and underscore the need for a more nuanced understanding of risk in this vulnerable population to inform policymaking.

Previous studies in England have documented substantial increases in all-cause and COVID-19-related mortality during the first (1^st^ February 2020 to 31^st^ August, 2020), but not the second (1^st^ September 2020 to 31^st^ March 2021) wave of the pandemic in residents relative to community-dwelling adults of comparable age [5], and a further reduction in COVID-19-related mortality IFR following the emergence of the Omicron variant in November 2021 [16]. Our findings regarding the decline in IFRs in residents over successive waves of the pandemic are similar to those reported by Ontario's public health agency [17]. Interestingly, the IFR reported in this study for COVID-19 in the first quarter of 2022 (6.9%) and onwards is similar to the median IFR (6.5%) for influenza in residents [18]. Restrictions on visiting care homes were withdrawn on 31^st^ January 2022 [19] but regular asymptomatic testing for SARS-CoV-2 in care home staff remained until 31^st^ August 2022 [20] and the requirement to wear facemasks in care homes remained until 15^th^ December 2022 [10]. Before the COVID-19 pandemic, NPIs were not routinely deployed in care homes during influenza season to prevent the spread of respiratory infection, unless there was an outbreak.

Although deaths attributable to SARS-CoV-2 in residents declined substantially over the study period, the IR of COVID-19-related mortality in residents in this study exceeded that seen in adults with severe comorbidities, who were prioritized for treatment with antivirals and neutralizing monoclonal antibodies (nMAb) to reduce their risk of severe outcomes. For example, our IR estimates of COVID-19-related mortality in residents during the second (0.26–0.47 per 1 kpd person days, Q4 2020 to Q1 2021) and third (0.02–0.07 per 1 kpd, Q2–Q4 2021) waves of the pandemic were substantially higher than those reported in the population-based OpenSAFELY study [21], which estimated COVID-19 mortality in patients with severe comorbidities such as Stage 5 Chronic Kidney Disease (0.18 and 0.04 per 1 kpd in the second (September 2020 to April 2021) and third (May 2020 to December 2020) waves, respectively) and haematological malignancies (0.04 and 0.018 per 1 kpd). Whilst it is difficult to directly compare estimates derived from the general population with care home residents because of the strong association between age and mortality [22], these findings underscore the extreme vulnerability of residents to severe outcomes following infection with SARS-CoV-2, and suggest they may benefit from access to antivirals and/or mAbs if these can be safely administered in care settings.

This study benefited from access to data from the UK's SARS-CoV-2 national screening programme. This facilitated reliable linkage of residents to care homes and estimation of individual time at risk. In contrast to other mortality analyses that have reported on symptomatic infections alone [6], we were able to include both asymptomatic and symptomatic infections, therefore optimizing data capture and allowing for less biased estimation of the IFR. However, the testing programme was only established in the summer and autumn of 2020, and thus it was not possible to investigate mortality incidence or IFRs in Wave 1 of the pandemic. It is also likely that we have underestimated the impact of prior infection on IFR as many infections occurring in the first half of 2020 were not recorded due to limited testing access over this period.

Although we present data on hospital admissions associated with SARS-CoV-2, we have focused on mortality linked to the virus. This is because hospital admission can be influenced by non-clinical factors such as Accident and Emergency waiting times [23] and chronic and acute problems in bed capacity and staffing in the context of an overstretched healthcare system [24,25]. There are signs of such effects in our data. The first quarter of 2021 was the peak of the Alpha variant wave and a period of extreme pressure on the functioning of hospitals in the UK; within our data, we observed the highest IFR of 24.9% in this period. However, the observed IHR of 12.1% was lower than the values observed in the two subsequent quarters (after widespread primary vaccination) and lower than that in Q3 2022 (12.5%), by which point the IFR had dropped to 8.1%.

A limitation of our analysis is that inclusion in our cohort of residents is dependent on linkage of individuals to participating care homes through reported SARS-CoV-2 tests. Regular asymptomatic testing of residents was discontinued at the end of March 2022, and thus residents with admission to the care homes beyond this point may have been omitted from the cohort because of a lack of SARS-CoV-2 testing. A further limitation of this study is that we were unable to access reliable data on co-morbidities and ethnicity, both factors widely associated with mortality, therefore we could not account for these in our analysis.

Our study demonstrates the use of routinely collected mortality data to monitor the impact of SARS-CoV-2 in care homes over a period of rapidly changing policy and evolution of circulating viral variants. Such approaches could be of immense value for public health teams to support more nuanced decisions on the escalation and de-escalation of NPIs in frail and comorbid populations with high baseline mortality such as care home residents, provided the data are available in near real-time. We used data on infection and mortality, capitalizing on the SARS-CoV-2 testing infrastructure that was established during the pandemic to identify residents, but there is scope to harness other sources of routinely collected data to inform the public health management of other, common infections in care home residents such as influenza or norovirus, that cause major outbreaks every year. This could be achieved through data linkage, for example by incorporating information on hospital admissions, microbiology and virology, outbreaks and even prescribing data, provided there is a reliable method to identify care home residents in routinely collected data. Importantly, analysis of routine data cannot address the urgent need for carefully designed research studies to evaluate the benefits and harms of NPIs in care homes.

## Conflict of interest statement

L.S. reports grants from the Department of Health and Social Care during the conduct of the study and is a member of the Social Care Working Group, which reports to the Scientific Advisory Group for Emergencies. A.H. reports funding from the COVID Core Studies Programme and is a member of the New and Emerging Respiratory Virus Threats Advisory Group at the Department of Health and Environmental Modelling Group of the Scientific Advisory Group for Emergencies. All other authors declare no competing interests.

## Funding sources

This work is independent research funded by the Department of Health and Social Care (COVID-19 surveillance studies). M.K. is funded by a Wellcome Trust Clinical PhD Fellowship (222907/Z/21/Z). L.S. is funded by a National Institute for Health and Care Research Professorship (302435). A.H. is supported by Health Data Research UK (LOND1), which is funded by the UK Medical Research Council, Engineering and Physical Sciences Research Council, Economic and Social Research Council, Department of Health and Social Care (England), Chief Scientist Office of the Scottish Government Health and Social Care Directorates, Health and Social Care Research and Development Division (Welsh Government), Public Health Agency (Northern Ireland), British Heart Foundation, and Wellcome Trust. The funders did not have a role in the design, execution, analysis and interpretation of data, or writing of this study.
