# Supplementary material for: COVID-19-related mortality and hospital admissions in the VIVALDI study cohort: October 2020 to March 2023
Source: J Hosp Infect. 2024 Jan;143:105–12. doi: 10.1016/j.jhin.2023.10.021 (PMC10927615; doi:10.1016/j.jhin.2023.10.021)
Supplement: Multimedia component 1 [file mmc1.docx]

**Supplementary materials**

**Figure S1** Flow chart of inclusion of older care home residents in the overall analysis, and for the evaluation of the infection fatality ratio for SARS-CoV-2.

|  |  | Excluded |  |
| --- | --- | --- | --- |
| Total participant records | 73059 |  |  |
|  | **↓** | 9402 | No PCR or LFD results (at any time) |
|  | 63657 |  |  |
|  | **↓** | 263 | Missing demographic data |
|  | 63394 |  |  |
|  | **↓** | 33824 | Resident<65, or staff |
|  | 29570 |  |  |
|  | **↓** | 1030 | Death before analysis start date |
|  | 28540 |  |  |
|  | **↓** | 2154 | Not in Vivaldi home in analysis period |
|  | 26386 |  |  |
|  | **↓** | 90 | Missing one or more vaccine date |
|  | 26296 |  |  |
|  | **↓** | 10 | Death on first day of at-risk period |
| Total participants | 26286 |  |  |
|  | **↓** | 17773 | No +ve test within analysis period |
| Residents with positive SARS-CoV-2 test in analysis period | 8513 |  |  |

**Figure S2** Plot of the number of residents considered to be under follow-up within the VIVALDI cohort over the analysis period for this study. Residents join the cohort at first recorded PCR or LFD test within a participating care home and exit the cohort at the earliest of: 90 days after last test linked to participating home if prior to 1^st^ January 2022, 31^st^ March 2023 if last test recorded on or after 1^st^ January 2022, or date of death.


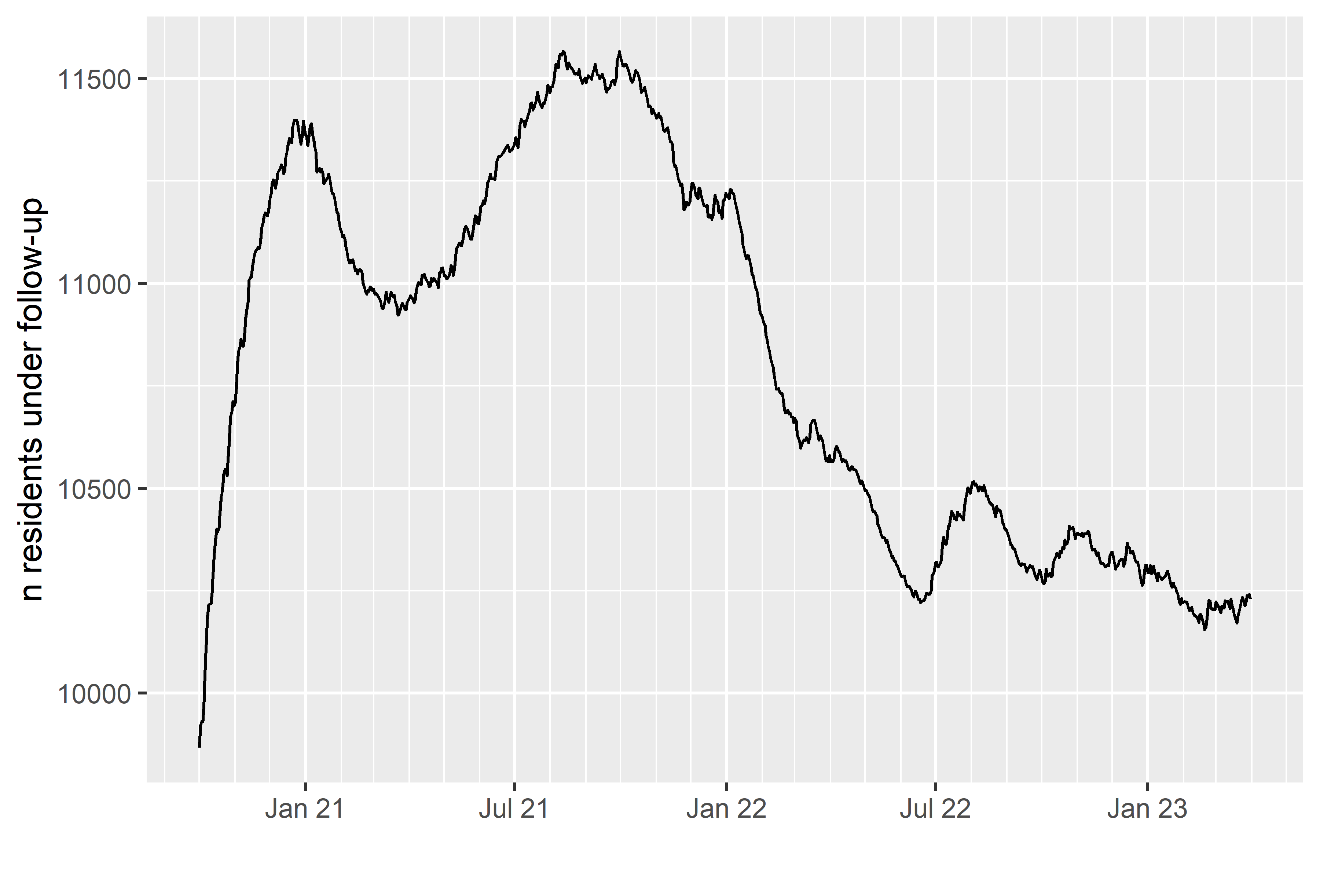


**Figure S3** Estimated incidence rates (IR) per 1000 person-days (1kpd) for overall mortality (black points) and COVID-19 related mortality (red points) among residents of long term care facilities within the VIVALDI study cohort, by calendar quarter. Errors bars show 95% CIs.


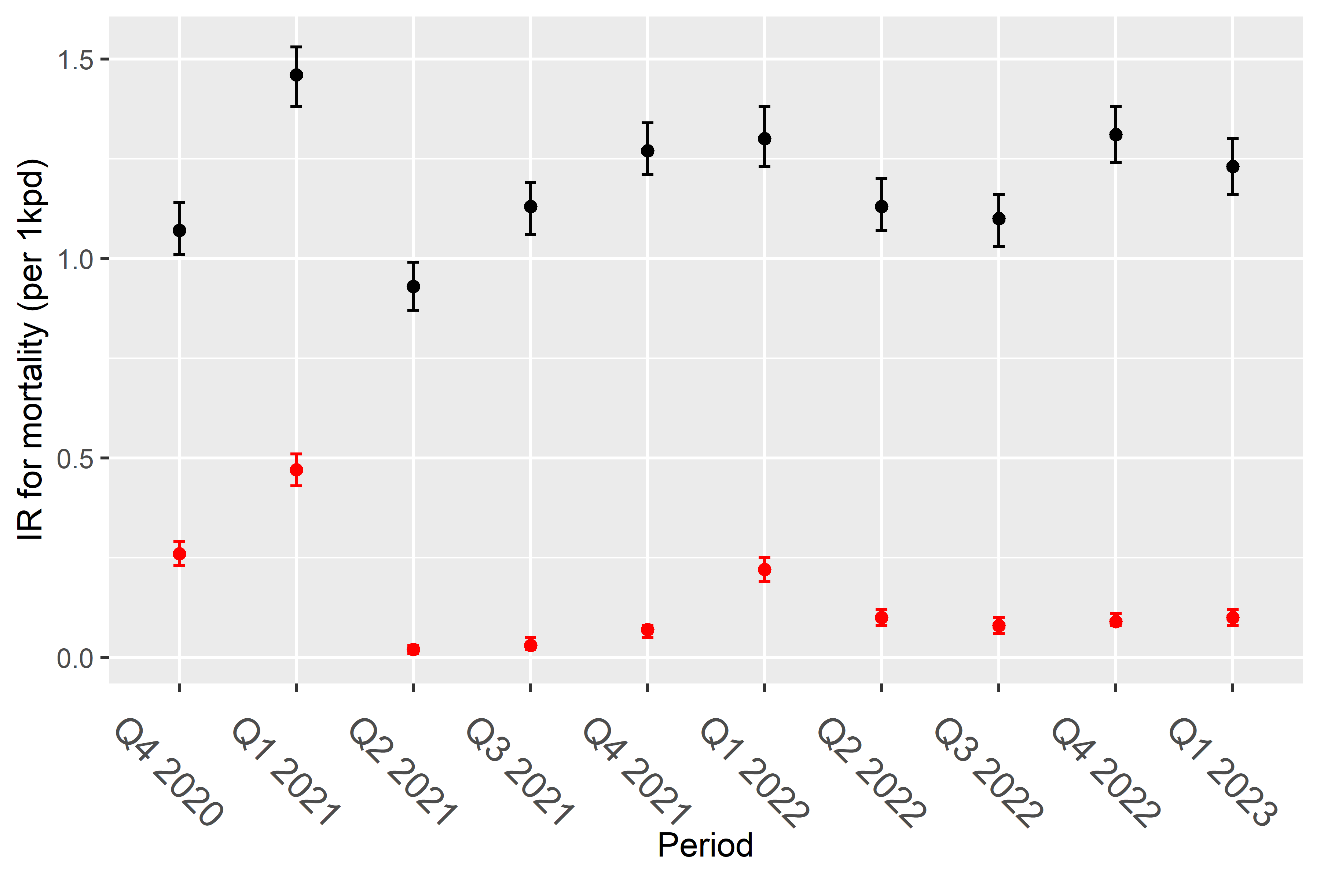


**Table S1** Results of Cox regression analyses of hospitalisation in the 14 days following a positive PCR or LFD test for SARS-CoV-2. Both models include a variable describing vaccination status, broken down by time since second dose or most recent booster dose received at point of positive test. Model 2 also includes a separate variable recording whether each person had received a bivalent SARS-CoV-2 vaccine prior to positive test.

|  | **Model 1,**  **HR (95%CI)** | **Model 2,**  **HR (95%CI)** |
| --- | --- | --- |
| Age (years) | 1.00 (0.99-1.00) | 1.00 (0.99-1.00) |
| Female | REF | REF |
| Male | 1.69 (1.48-1.93) | 1.69 (1.48-1.93) |
| Calendar period |  |  |
| Q4 2020 | REF | REF |
| Q1 2021 | 1.02 (0.78-1.33) | 1.02 (0.77-1.33) |
| Q2 2021 | 2.01 (0.83-4.84) | 1.97 (0.82-4.78) |
| Q3 2021 | 1.69 (0.96-2.96) | 1.65 (0.94-2.90) |
| Q4 2021 | 1.16 (0.75-1.80) | 1.12 (0.72-1.74) |
| Q1 2022 | 0.78 (0.52-1.17) | 0.74 (0.49-1.12) |
| Q2 2022 | 1.33 (0.86-2.07) | 1.29 (0.83-2.01) |
| Q3 2022 | 1.45 (0.93-2.25) | 1.40 (0.90-2.18) |
| Q4 2022 | 0.94 (0.60-1.47) | 1.09 (0.68-1.75) |
| Q1 2023 | 1.07 (0.68-1.68) | 1.28 (0.78-2.09) |
| *Vaccination status* |  |  |
| None | REF | REF |
| Single vaccine dose | 0.61 (0.43-0.87) | 0.62 (0.43-0.88) |
| 2-12 weeks since D2 | 0.71 (0.33-1.50) | 0.72 (0.34-1.53) |
| 12-24 weeks since D2 | 0.73 (0.42-1.28) | 0.75 (0.43-1.31) |
| 24+ weeks since D2 | 0.67 (0.44-1.02) | 0.69 (0.45-1.05) |
| 2-12 weeks since booster | 0.58 (0.39-0.85) | 0.61 (0.42-0.91) |
| 12-24 weeks since booster | 0.62 (0.43-0.89) | 0.66 (0.45-0.95) |
| 24+ weeks since boost | 0.69 (0.46-1.03) | 0.69 (0.46-1.02) |
| Prior infection | 0.84 (0.70-1.01) | 0.84 (0.70-1.01) |
| Bivalent vaccine received |  | 0.74 (0.53-1.05) |

HR, hazard ratio.
